# Molecular Characterization of Vancomycin-Resistant *Enterococcus* spp. from Clinical Samples and Identification of a Novel Sequence Type in Mexico

**DOI:** 10.3390/antibiotics14070663

**Published:** 2025-06-30

**Authors:** Raúl Alejandro Atriano Briano, Nallely S. Badillo-Larios, Perla Niño-Moreno, Luis Fernando Pérez-González, Edgar A. Turrubiartes-Martínez

**Affiliations:** 1 Laboratory of Hematology, Faculty of Chemical Sciences, Autonomous University of San Luis Potosi, San Luis Potosi 78240, Mexico; raul.atriano@uaslp.mx; 2 Center of Research in Health Sciences and Biomedicine, Faculty of Medicine, Autonomous University of San Luis Potosi, San Luis Potosi 78210, Mexico; nallely.sarai@gmail.com (N.S.B.-L.);; 3 Genetics Laboratory, Faculty of Chemical Sciences, Autonomous University of San Luis Potosi, San Luis Potosi 78240, Mexico; 4 Faculty of Medicine, Autonomous University of San Luis Potosi, San Luis Potosi 78240, Mexico; 5 High Specialty Regional Hospital “Dr. Ignacio Morones Prieto”, San Luis Potosi 78240, Mexico

**Keywords:** *Enterococcus*, multidrug resistance, antibiotic resistance, vancomycin-resistant enterococcus, virulence factors, multilocus sequence typing, ST2700 *vanA*

## Abstract

**Background:***Enterococcus* spp. is the third leading cause of healthcare-associated infections in the American continent, often because of the virulence factors that protect the bacterium against host defenses and facilitate tissue attachment and genetic material exchange. In addition, vancomycin, considered a last-resort treatment, has shown reduced efficacy in *Enterococcus* spp. strains. However, the relationship between bacterial resistance and virulence factors remains unclear. This study intends to evaluate the prevalence of glycopeptide-resistant genotypes and virulence factors in *Enterococcus* spp. strains. **Methods:** Over six months, 159 *Enterococcus* spp. strains causing nosocomial infections were analyzed. Multiplex PCR was performed to identify species, glycopeptide-resistant genotypes, and 12 virulence factors. **Results:** The most abundant species identified were *Enterococcus faecalis* and *E. faecium*. Vancomycin resistance was observed in 10.7% of the isolates, and the *vanA* genotype was present in 47% of resistant samples. The main virulence factors detected were *acm* (54%), which is related to cell adhesion; gel E (66%), a metalloproteinase linked to tissue damage; and the sex pheromones *cpd* (64%) and *ccf* (84%), which are involved in horizontal gene transfer. A significant association was found between the prevalence of *acm*, *ccf*, and *cpd* in VRE isolates, indicating the potential dissemination of genes to emerging strains via horizontal gene transfer. In addition, a new *E. faecium*, which displayed five virulence factors and harbored the *vanA* sequence type, was identified and registered as ST2700. **Conclusions:** *Enterococcus faecalis* and *E. faecium* are clinically critical due to multidrug resistance and virulence factors like *acm*, which aids host colonization. Genes *ccf* and *cpd* promote resistance spread via horizontal transfer, while the emerging ST2700 strain requires urgent monitoring to curb its virulent, drug-resistant spread.

## 1. Introduction

Globally, *Enterococcus faecalis* and *Enterococcus faecium* are opportunistic pathogens responsible for healthcare-associated infections (HAIs), which can cause urinary tract infections, surgical wound infections, bacteremia, and endocarditis [1,2]. According to the Centers for Disease Control and Prevention (CDC) and the European Center for Disease Prevention and Control (ECDC), they are the third leading cause of HAIs in America and the fifth leading cause in Europe [3,4].

Treating infections caused by *Enterococcus* spp. has become more challenging because they are intrinsically resistant to cephalosporins, trimethoprim/sulfamethoxazole, and therapeutic concentrations of aminoglycosides and clindamycin [5,6]. The CDC estimates that 30% of infections caused by *Enterococcus* spp. in the United States are resistant to vancomycin (VRE). In Mexico, studies report a VRE prevalence as high as 21% [2,7,8]. Nine different genotypes of resistance to glycopeptides (*vanA*, *vanB*, *vanC*, *vanD*, *vanE*, *vanG*, *vanL*, *vanM*, and *vanN*) have been reported, resulting in chromosomal mutations or the acquisition of mobile genetic elements. Among these, *vanA* and *vanB* stand out in nosocomial outbreaks with multidrug-resistant (MDR) strains implicated in several infections, especially in immunocompromised patients [5,8,9].

Multilocus sequence typing (MLST) has been applied in multiple international studies to investigate the epidemiology of VRE [10]. High recombination rates and the acquisition of mobile elements in the genome of *Enterococcus* spp. confirms a large collection of strains in Latin America [11]. MLST analyses different sequences with single-locus variation at a particular locus, given a unique allele number and the combination of alleles in the seven housekeeping genes, and generates a sequence type (ST) for each isolate. The emergence of new variants reflects the alarming adaptability of *Enterococcus* spp. in hospital environments [10]. For example, the ST78 *vanA* lineage has spread worldwide and is described as one of the main genotypes of VRE with the potential to emerge as a successful clone [12,13]. Additionally, ST28, which possesses multiple virulence factors, has been identified as a high-risk multidrug-resistant strain with potential implications for public health [14,15].

On the other hand, virulence factors contribute to enterococcal fitness and its persistence as a pathogen, especially in the nosocomial environment [16,17]. They include enzymes that enable the hydrolysis of collagen, casein, hemoglobin, hyaluronic acid, erythrocytes, and bacterial cells (e.g., gelatinase, hyaluronidase, and cytolysins) [18,19]; surface-associated proteins that anchor to the bacterial cell wall and facilitate physical contact with host cells (e.g., aggregation substances, enterococcal surface protein, and collagen adhesin); and factors that promote bacterial conjugation and the acquisition of new genes (e.g., sex pheromones) [20,21,22].

In this context, infections caused by *Enterococcus* spp. present two main challenges, intrinsic resistance to commonly used antibiotics [3] and virulence factors, which can facilitate colonization, the invasion of host tissue, and the evasion of the immune response [1,4,5]. Previous research such as that of Ghaziasgar et al. and Arshadi et al. has reported associations between certain virulence factors and glycopeptide resistance or multidrug resistance [23,24,25,26]; however, this relationship between virulence and resistance remains unclear [9,11].

Therefore, the present investigation aimed to elucidate the potential association between the virulence and the resistance genotype to glycopeptides (vancomycin) in enterococcal isolates.

## 2. Results

### 2.1. Clinical Characteristics of Patients and Clinical Isolates

A total of 159 clinical isolates of *Enterococcus* spp. were collected. The samples were mainly obtained from male patients aged less than 1 year to 90 years. The strains were collected mostly from secretions (*n* = 88, 55.3%) and urine (*n* = 44, 27.7%). *E. faecalis* was the most frequently isolated species (*n* = 119, 74.8%), followed by *E. faecium* (*n* = 22, 13.8%), *E. gallinarum* (*n* = 6, 3.8%), and *E. casseliflavus* (*n* = 3, 1.9%). Surgery was the hospital service with the most strains collected (*n* = 49, 30.8%), and *E. faecium* had higher prevalence than that other *Enterococcus* species (OR = 4.05, *p* = 0.02) (Table 1).

### 2.2. Antimicrobial Susceptibility

Table 2 shows antimicrobial susceptibility profiles of samples analyzed in this study. Eighty-five isolates (53.4%) were MDR, and 17 (20%) were vancomycin resistant. More than 30% of isolates were resistant to quinolones and gentamicin. Additionally, four isolates resistant to linezolid (2.5%) were identified. *E. faecium* presented a profile with significantly higher multidrug resistance than that of the other species (*p* = 0.00). Prevalence of *E. faecium* strains resistant to ampicillin, penicillin G, ciprofloxacin, levofloxacin, and high-level streptomycin was higher compared with the other species. Additionally, seven isolates (27.3%) were resistant to vancomycin, which represents a higher prevalence than that of the other species (*p* = 0.00).

From 17 VRE isolates, the *vanC* genotype was identified in 9 isolates. The *vanA* genotype was the second most prevalent present in eight isolates. Finally, the *vanB* genotype was not identified in any isolate (Table 3).

### 2.3. Virulence Factors

Twelve virulence factors were analyzed in the present study using multiplex PCR, including five secretion products (*hyl*, *gel E*, *cylA*, *cylB*, and *cylM*), four factors related to adhesion to host tissues (*acm*, *asa*, *esp*, and *agg*), and three factors that facilitate bacterial conjugation (*ccf*, *cpd*, and *cob*). As indicated in Table 4, the virulence genes *ccf*, *gel E,* and *cpd* showed higher prevalence (*n* = 135, 84.9%; *n* = 105, 66%; and *n* = 103, 64.8%, respectively). Interestingly, the virulence factors *cylA*, *agg*, *cylB*, and *cylM* were exclusively found in *E. faecalis*. The virulence genes *ccf* (*n* = 108, 90.8%), *cpd* (*n* = 94,79%), *gel E* (*n* = 87, 73.1%), and *asa* (*n* = 70, 58.8%) were significantly higher in *E. faecalis* than in the other species analyzed (*p* < 0.05).

*E. faecalis* exhibited higher rates of virulence factors than other species. Specifically, this species presented a significant difference in the prevalence of *cylA*, *cylB*, *cylM*, *gel E*, and *hyl*) (*p* = 0.001) and *ccf*, *cpd*, and *cob* (*p* = 0.006) compared to other *Enterococcus* species. Additionally, the same difference was found in *E. faecium*, which is more likely to have an adhesion virulence factor than other species (Table 5). No significant differences were observed in the distribution of virulence factors among different infection types.

As shown in Table 6, more than half of the strains were characterized as MDR (including resistance to vancomycin), and the possible relationship between this characteristic and virulence factors was studied. The data obtained showed that *Enterococcus* spp. MDR strains were more likely to have *acm* (*p* < 0.05) and less likely to have *gel E* (*p* < 0.05). Moreover, VRE isolates tended to have the *acm* virulence gene compared to VSE strains. In contrast, vancomycin resistance tended to be absent in strains that possessed *ccf* and *cpd* (*p* = 0.05).

Regarding the relationship between the glycopeptide resistance genotype and virulence genes, a high number of VRE *vanA* genotype strains harbored *acm* but were less likely to carry *gel E* and *cpd* (*p* = 0.05). Conversely, *vanC* genotype strains exhibited a lower probability of owning *ccf* than *Enterococcus* spp. strains without this genotype (*p* = 0.05) (Table 7).

### 2.4. MLST

Exploring the clinical data, the strain denominated as B630 resulted in MDR *Enterococcus* spp. (including vancomycin) involved in a polybacterial infection with another MDR bacterial (*Pseudomonas aeruginosa*) strain in a patient with a long-term hospital stay (234 days) and multiple surgeries due to infection. After this discovery, molecular analysis was performed, resulting in the finding that this strain harbored the highest number of genes related to virulence factors (five) and carried the *vanA* genotype, which was the reason for its selection for MLST. After sequencing, a new allele for *gyd* was found and registered in the Pub MLST under the ID: BIGSdb_20240713023858_976194_47037; with this new sequence, a new sequence type (ST2700) was identified and approved under ID:BIGSdb_20240715212428_2553542_15385.

## 3. Discussion

In recent years, infections caused by *Enterococcus* spp., especially VRE strains, have become a global health burden [26]. Some of the reasons for this include the plasticity of its genome, which allows rapid adaptability to adverse environments by acquiring genetic determinants that increase its ability to colonize or infect the host [27]. In Mexico, data on enterococcal infections are scarce [28]. Therefore, this study aimed to determine the prevalence of glycopeptide resistance and virulence factors in clinical strains of *Enterococcus* spp.

In the present study, *E. faecalis* was the most prevalent species, followed by *E. faecium*. Similar data have been reported in other studies, which mention that the main species of *Enterococcus* associated with hospital infections are *E. faecalis* followed by *E. faecium*; together, they cause approximately 90% of infections [21,29,30].

The *Enterococcus* genus is characterized by its ability to survive in adverse conditions and its adaptability in hospital environments [6,31,32]. Postoperative enterococcal infections have been associated with prior exposure to antibiotics such as cephalosporins and ampicillin, primarily administered as a prophylaxis [33]. In this study, *E. faecium* was more likely to be isolated from surgical samples than other species of the same genus. Similar data were reported by Pochhammer et al., where *E. faecium* was associated with 70% of surgical site infections [34]. *Enterococcus* spp. infections after surgical treatment are an important cause of morbidity and mortality, which raises the need to implement strategies to prevent and identify the causal agent of infection [35].

The unjustified use of antibiotics has increased the incidence of MDR *Enterococcus* spp. strains [36]. In the present study, 53.4% of the isolates were MDR; similar data have been reported by Phoon et al., Farman et al., and Mohanty et al., who reported a prevalence of 49–96% of *Enterococcus* spp. isolates with a multidrug resistance profile. These data reflect the alarming global problem of inefficiency in first-line treatments [37,38,39].

A common strategy for the treatment of enterococcal infections is the use of aminoglycosides and penicillin because of their synergy. In cases of MDR strains, the treatment is vancomycin, and for infections with vancomycin-resistant *Enterococcus* spp. strains, linezolid is the treatment of choice [36]. The present study found that 36.5%, 10.7%, and 2.5% of the strains were resistant to aminoglycosides, vancomycin, and oxazolidinones, respectively, which are the treatments for severe infections caused by *Enterococcus* spp. In a multicenter study conducted in Latin America, the prevalence of resistance to aminoglycosides and glycopeptides was 28% and 6%, respectively. In Mexico, the prevalence of resistance to aminoglycosides is estimated to be 29%, and to glycopeptides, 4–20%; however, these percentages refer to the most prevalent species in hospitals (*E. faecalis* and *E. faecium*) and do not consider the others [7,28,40,41]. On the other hand, resistance to linezolid was recently described in Mexico [42], and in this study, four *E. faecalis* isolates resistant to this antibiotic were identified.

In this research, data showed that *E. faecium* is more likely to be MDR and resistant to vancomycin (both, *p* = 0.001) than other species of the same genus, which is consistent with several worldwide studies [4,41,42,43,44]. According to the CDC, more than 70% of central line-associated bloodstream infections (CLABSIs) caused by *E. faecium* are resistant to vancomycin [45]. In 2021, the ECDC reported a 17.2% prevalence of vancomycin-resistant *E. faecium* [2]. These data indicate that MDR *E. faecium* in recent years is emerging as an important clinical pathogen.

A new ST of *E. faecium*, identified as ST2700, has been involved in a polybacterial infection with another multidrug-resistant bacterial, a holder of five virulence factors (*acm*, *asa*, *esp*, *gel E*, and *ccf*), and the *vanA* genotype (high resistance level). To elucidate if this strain belongs to a specific clone MLST, a method for surveillance and typing sequence types (STs) was performed. This technique has been applied in multiple international studies to investigate the epidemiology of VRE. High recombination rates and the acquisition of mobile elements in the *Enterococcus* spp. genome have been reported [10,11,46] with this technique. It has been determined that most of the clinically noteworthy *E. faecium* strains share a common ancestor linked with the clonal complex CC17, which is associated with hospital outbreaks. The emergence of new variants reflects the alarming adaptability of *Enterococcus* spp. to hospital environments [11,47]. For example, the ST78 *vanA* lineage has spread worldwide and is described as a high-risk, multidrug-resistant strain and one of the main STs of VRE with the potential to emerge as a successful clone [12,13,14,47]. In addition, new variants have emerged, such as *vanA* lineage ST1299 with multiple drug resistance which was initially identified in Germany and responsible for several hospital outbreaks in Austria in 2021, demonstrating its rapid spread [48].

Vancomycin resistance is explained by a mutation present in the terminal amino acid related to the production of the peptidoglycan of the bacterial wall. This resistance is considered high level when the precursor of the bacterial wall changes from D-Alanine-D-Alanine to D-Alanine-D-Lactate, it is linked to the *vanA* genotype, and presents a Minimum Inhibitory Concentration (MIC) higher than 32 μg/mL, or it is considered low level when linked with the *vanC* genotype (MIC 4-32 μg/mL) when the terminal amino acid changes to D-Alanine-D-Serin [5,36]. In this study, the predominant is the *vanC* genotype which differs from data reported in Germany, China, and Canada, where the *vanA* and *vanB* genotypes are predominant. Meanwhile, in South America, the *vanA* genotype predominates with 80.8% [9,36,41,49,50]. Most studies are focused on the *vanA* genotype, and data on the prevalence of the *vanC* genotype are scarce and restricted to epidemiological studies in specific populations. Britt NS et al. found therapeutic failure in 39.6% of cases of enterococcal bacteremia caused by non-*faecium*, and non-*faecalis* species; and 10.4% of deaths were associated with infections caused by species with the *vanC* genotype [51]. Coccitto and Marzia et al. report the presence of three genetic determinants of linezolid resistance in *E. gallinarum*, an intrinsically vancomycin-resistant strain [52]; this highlights that intrinsic resistance to vancomycin poses therapeutic challenges like those faced during the treatment of VRE strains with the *vanA/vanB* genotypes [53,54]. Microbiological and clinical studies focusing on the ecology and pathogenesis of *vanC* genotype strains are necessary in Mexico to control and manage infections caused by enterococcal species.

In addition to antimicrobial resistance, the presence of virulence factors in strains of *Enterococcus* spp. is another factor to consider for the severity of infections [31,49,55,56,57,58]. In the present research, virulence factors detected in more than 50% of the *Enterococcus* spp. isolates were *ccf*, *cpd*, and *gel E*. However, Jahansepas et al. and Heidari et al. report a high prevalence of the virulence factors *acm*, *gel E*, and *hyl* in clinical isolates. On the other hand, Aung et al. report that *asa*, *gel E*, and *esp* are the main virulence factors and indicate a wide diversity of virulence factors in *Enterococcus* spp. strains causing healthcare-associated infections [15,31,44].

The pathogenicity of *Enterococcus* spp. increases with virulence factors that allow it to adhere, invade, or immunosuppress host tissues [59]. In this study, *E. faecalis* was the most virulent strain compared to other species, presenting some secretion factors that facilitate conjugation; this aligns with the studies of Igbinosa et al. and Ghaziasgar et al., where *E. faecalis* presented more than three virulence factors, distributed in two groups [25,60]; this suggests that secretion and conjugation virulence factors potentially contribute to the establishment of the bacterium in host tissues, which may indicate the reason for its higher prevalence over other *Enterococcus* species [44].

Virulence factors were initially identified in MDR *E. faecalis* strains that caused nosocomial outbreaks in 1980 [61]. To date, the relationship between MDR and virulence factors remains unclear. The data obtained in our study showed a statistically significant association between the prevalence of the virulence factors *acm* and *gel E* in MDR strains compared to non-MDR strains. Research conducted by Shahram Shahraki et al. found that the prevalence of the genes *asa*, *gel E*, and *acm* in MDR strains was 69%, 55%, and 40%, respectively. Doss Sundai et al. reported that the main virulence gene detected was *gel E* (78%), followed by *asa* (75%) and *esp* (70%) [59,62]. Available information suggests that an increase in antimicrobial multidrug resistance correlates with an increase in virulence and may be associated with an increase in mortality [63]. However, such information is limited.

The prevalence of VRE strains producing various virulence factors is increasing [12,39,59]. In this study, VRE strains were more likely to have the gene *acm* (*p* < 0.05). Sinel et al. reported that the expression of *acm* (which plays an important role in the development of endocarditis) in *E. faecium* is favored by the presence of antibiotics [64,65]. In addition, it has been proven that in vitro, the protein encoded by the gene *acm* mediates binding with type I collagen, an important step in infections by this genus [63,66].

Some studies discuss the role of *ccf* and *cpd* in VRE. Hirt et al., in a murine model, found that sex pheromone-induced plasmid transfer occurs within 5 h of introducing donor strains into the environment of plasmid-free bacteria; this demonstrates the importance of sex pheromones in adaptability to new ecological niches [20]. Interestingly, studies suggest that plasmids susceptible to enterococcal pheromones do not necessarily harbor resistance genes, implying that they may encode other molecular traits related to the colonization of their host cells and the costs of plasmid maintenance [22,67].

## 4. Materials and Methods

### 4.1. Bacterial Isolates

Clinical isolates of non-repeated *Enterococcus* spp. causing HAIs were collected from the High Specialty Regional Hospital “Dr. Ignacio Morones Prieto” in the city of San Luis Potosí, San Luis Potosí, Mexico. The samples were collected between October 2018 and April 2019. This study was approved by the Research Committee [COFEPRIS 17 CI 24 028 093] and the Research Ethics Committee of the High Specialty Regional Hospital “Dr. Ignacio Morones Prieto” [CONBIOETICA-24-CEI-001-20160427] with the registration number 56-17. Informed consent was obtained from all participants or legal guardians.

Data collected from patient medical records included sex, age, length of hospital stay, and outcome. The reason for hospitalization differs from the identified infection was verified in that infection-related symptomatology was not present until a minimum of 48 h after the patient’s admission. This methodological approach was deliberately employed to mitigate classification bias and ensure accurate differentiation between community-acquired and hospital-acquired infections.

### 4.2. Bacterial Identification and Antimicrobial Susceptibility

Genus identification was performed using biochemical tests, growth in brain-heart infusion broth with 6.5% NaCl, and the hydrolysis of esculin in bile esculin agar. Antimicrobial susceptibility (including MIC) was assessed using VITEK^®^ 2 Compact semi-automated equipment (bioMérieux, Marcy-l’Étoile, France), following the specifications of the Clinical and Laboratory Standards Institute (CLSI), using the following antibiotics: vancomycin (resistance ≥ 32 µg/mL), ampicillin (resistance ≥ 16 µg/mL), benzylpenicillin (resistance ≥ 16 µg/mL), ciprofloxacin (resistance ≥ 4 µg/mL), levofloxacin (resistance ≥ 8 µg/mL), streptomycin (resistance > 1000 µg/mL), gentamicin (resistance > 500 µg/mL), erythromycin (resistance ≥ 8 µg/mL), tetracycline (resistance ≥ 16 µg/mL), linezolid (resistance ≥ 8 µg/mL), Quinupristin/Dalfopristin (resistance ≥ 4 µg/mL), and Tigecycline (resistance ≥ 0.12 µg/mL). 

### 4.3. Bacterial DNA Extraction

Bacterial DNA was collected from fresh colonies using the Wizard ^®^ Genomic DNA Purification Kit (Promega, San Luis Obispo, CA, USA), according to the manufacturer’s specifications. The obtained DNA was quantified using an OPTIZEN™ nanobiophotometer (K Lab Co., Ltd., Gunpo-si, Republic of Korea) to evaluate its purity and concentration.

### 4.4. Molecular Confirmation of Species and Glycopeptide Resistance Genotype

For the molecular identification of *Enterococcus* species and identifying glycopeptide resistance genotypes, a multiplex PCR was performed using 100 ng of bacterial DNA with 0.2–0.4 μM of each oligonucleotide for the species *E. faecalis*, *E. faecium*, *E. gallinarum*, and *E. casseliflavus*, and for resistance genotypes *vanA*, *vanB*, and *vanC* [25] (Table S1). Reactions were carried out in 1× PCR buffer, 4 mM of MgCl_2_ and 2 mM of deoxynucleotide triphosphates (dNTPs), with 1 unit of Taq DNA polymerase (Expand High Fidelity, Taq Polymerase, Roche Applied Science, Penzberg, Germany) in 25 μL reactions. The PCR conditions were as follows: initial denaturation at 94 °C for 5 min, followed by 30 cycles of 94 °C for 1 min, 55 °C for 1 min, and 72 °C for 1 min, with a final extension at 72 °C for 10 min.

The products were separated on 2% agarose gels in 1× Tris-boric acid-EDTA (TBE) buffer and visualized with ethidium bromide using a UV light photodocumenter [68,69].

### 4.5. Molecular Detection of Virulence Factors

Two multiplex PCRs were standardized for the identification of virulence factors: the first included the genes *cylA*, *gel E*, *hyl*, *asa*, *esp*, and *acm*, and the second included the genes *cylB*, *cylM*, *agg*, *ccf*, *cpd,* and *cob*. Both reactions were carried out with 1X PCR buffer, 3 mM of MgCl_2_, 0.4 μM of each oligonucleotide, and 2 mM of deoxynucleotide triphosphates (dNTPs), with 1 unit of Taq DNA polymerase (Expand High Fidelity, Taq Polymerase, Roche Applied Science) in 25 μL reactions. The PCR conditions were as follows: initial denaturation at 94 °C for 5 min, followed by 35 cycles of 94 °C for 1 min, 50 °C or 52 °C for 1 min, and 72 °C for 1 min, with a final extension at 72 °C for 7 min.

The products were separated in 2% agarose gels in 1× Tris-boric acid-EDTA (TBE) buffer and visualized with ethidium bromide using a UV light photodocumenter [68,69].

### 4.6. Multilocus Sequence Typing (MLST)

The isolate named B630 of *Enterococcus faecium* was designated for analysis by MLST, which was performed with a set of primers and PCR procedures, and sequenced as described by Homan et al., which amplified the seven housekeeping genes adk, atpA, ddl, gyd, gdh, purK, and pstS [70].

The PCR products sequenced were compared with the MLST database (https://pubmlst.org/bigsdb?db=pubmlst_efaecium_isolates accessed on 16 July 2024) to determine the sequence types (STs) of the isolates. 

### 4.7. Statistical Analysis

The associations between the virulence factors analyzed and antimicrobial susceptibility were examined with GraphPad Prism software (V.5, San Diego, CA, USA) using 2 × 2 contingency tables and Fisher’s exact test. The possible joint association of two or more variables with antimicrobial resistance was analyzed by multiple binary logistic regression using the SPSS software (ver.22, Chicago, IL, USA). Statistical significance was set at *p* < 0.05.

## 5. Conclusions

The diversity of virulence factors and their multidrug resistance profile have allowed *E. faecalis* and *E. faecium* to establish themselves as clinically important pathogens. The *acm* virulence gene in MDR isolates, particularly in VRE strains, suggests that it plays an important role in the colonization of host tissues, which could complicate treatment. Additionally, it should be noted that the prevalence of virulence factors *ccf* and *cpd*, which are related to bacterial conjugation, predisposes the diffusion of resistance genes to new strains through horizontal gene transfer.

Moreover, the ST2700 discovered in this study should be monitored to prevent the spread of this VRE, multidrug-resistant, and highly virulent strain.

## Figures and Tables

**Table 1:** Demographic data of patients infected with *Enterococcus* spp.

Species												
Demographic Data	*E. faecalis* 119 (74.8%)		*E. faecium* 22 (13.8%)		*E. gallinarum* 6 (3.8%)		*E. casseliflavus* 3 (1.9%)		Others 9 (5.7%)		Total 159 (100%)	
Sex												
Female	62	(52.1)	9	(40.9)	1	(16.7)	1	(33.3)	6	(66.7)	79	(49.7)
Male	57	(47.9)	13	(59.1)	5	(83.3)	2	(66.7)	3	(33.3)	80	(50.3)
Age range												
Newly born	12	(10.1)	3	(13.6)	0	(0.0)	0	(0.0)	1	(11.1)	16	(10.1)
Infants	9	(7.6)	0	(0.0)	0	(0.0)	0	(0.0)	0	(0.0)	9	(5.7)
Children	0	(0.0)	1	(4.5)	0	(0.0)	0	(0.0)	0	(0.0)	1	(0.6)
Adolescents	4	(3.4)	0	(0.0)	0	(0.0)	0	(0.0)	0	(0.0)	4	(2.5)
Young adult	7	(5.9)	2	(9.1)	1	(16.7)	2	(66.7)	0	(0.0)	12	(7.5)
Adult	59	(49.6)	14	(63.6)	4	(66.7)	1	(33.3)	6	(66.7)	84	(52.8)
Seniors	28	(23.5)	2	(9.1)	1	(16.7)	0	(0.0)	2	(22.2)	33	(20.8)
Ward												
Surgery	37	(31.1)	11	(50.0) *	0	(0.0)	0	(0.0)	1	(11.1)	49	(30.8)
Urgency	21	(17.6)	3	(13.6)	1	(16.7)	1	(33.3)	4	(44.4)	30	(18.9)
Medicine	15	(12.6)	2	(9.1)	0	(0.0)	0	(0.0)	1	(11.1)	18	(11.3)
External	16	(13.4)	0	(0.0)	1	(16.7)	0	(0.0)	1	(11.1)	18	(11.3)
Orthopedics	10	(8.4)	2	(9.1)	1	(16.7)	1	(33.3)	0	(0.0)	14	(8.8)
Gynecology	6	(5.0)	1	(4.5)	0	(0.0)	0	(0.0)	0	(0.0)	7	(4.4)
Infant	6	(5.0)	1	(4.5)	0	(0.0)	0	(0.0)	0	(0.0)	7	(4.4)
Burned	2	(1.7)	1	(4.5)	0	(0.0)	1	(33.3)	0	(0.0)	4	(2.5)
ICU	6	(5.0)	1	(4.5)	3	(50.0)	0	(0.0)	2	(22.2)	12	(7.5)
Specimen												
Body fluids	63	(52.9)	14	(63.6)	1	(16.7)	3	(100.0)	7	(77.8)	88	(55.3)
Urine	38	(31.9)	5	(22.7)	1	(16.7)	0	(0.0)	0	(0.0)	44	(27.7)
Biopsy	13	(10.9)	0	(0.0)	1	(16.7)	0	(0.0)	2	(22.2)	16	(10.1)
Blood	3	(2.5)	2	(9.1)	1	(16.7)	0	(0.0)	0	(0.0)	6	(3.8)
Catheter	1	(0.8)	1	(4.5)	0	(0.0)	0	(0.0)	0	(0.0)	2	(1.3)
Others	1	(0.8)	0	(0.0)	2	(33.3)	0	(0.0)	0	(0.0)	3	(1.9)

ICU, intensive care unit. * Odds Ratio: 4.05; CI: Confidence interval 95%: 1.6–10.3, *p* = 0.02.

**Table 2:** Antimicrobial susceptibility profiles of *Enterococcus* spp. isolates.

		*E. faecalis* 119 (74.8%)			*E. faecium* ^a^ 22 (13.8%)			*E. gallinarum* 6 (3.8%)			*E. casseliflavus* 3 (1.9%)			Others 9 (5.7%)		
Antibiotic / Interpretive Criteria	Total 159 (%)	S	I	R	S	I	R	S	I	R	S	I	R	S	I	R
VAN	17 (10.7)	118 (99.2)	0 (0)	1 (0.8)	15 (68.2)	0 (0)	7 (31.8) *	0 (0)	0 (0)	6 (100)	0 (0)	0 (0)	3 (100)	9 (100)	0 (0)	0 (0)
AMP	22 (13.8)	112 (94.1)	0 (0)	7 (5.9)	7 (31.8)	0 (0)	15 (68.2) *	6 (100)	0 (0)	0 (0)	3 (100)	0 (0)	0 (0)	9 (100)	0 (0)	0 (0)
PG	34 (21.4)	101 (84.9)	0 (0)	18 (15.1)	9 (40.9)	0 (0)	13 (59.1) *	6 (100)	0 (0)	0 (0)	3 (100)	0 (0)	0 (0)	6 (66.7)	0 (0)	3 (33.3)
CIP	56 (35.2)	78 (65.6)	1 (0.8)	40 (33.6)	4 (18.2)	3 (13.6)	15 (68.2) *	4 (66.6)	1 (16.7)	1 (16.7)	3 (100)	0 (0)	0 (0)	9 (100)	0 (0)	0 (0)
LVX	55 (34.6)	79 (66.4)	0 (0)	40 (33.6)	6 (27.3)	2 (9.1)	14 (63.6) *	5 (83.3)	0 (0)	1 (16.7)	3 (100)	0 (0)	0 (0)	9 (100)	0 (0)	0 (0)
HLS	23 (14.5)	103 (86.5)	0 (0)	13 (11.0)	15 (68.2)	0 (0)	7 (31.8) *	4 (66.7)	0 (0)	2 (33.3)	2 (66.6)	0 (0)	1 (33.3)	9 (100)	0 (0)	0 (0)
HLG	58 (36.5)	74 (62.2)	0 (0)	45 (37.8)	12 (54.5)	0 (0)	10 (45.5)	5 (66.7)	0 (0)	3 (33.3)	3 (100)	0 (0)	0 (0)	8 (88.9)	0 (0)	1 (1.1)
ERY	91 (57.2)	13 (10.9)	40 (33.6)	66 (55.4) *	1 (4.5)	1 (4.5)	20 (90.9)	6 (66.7)	0 (0)	4 (33.3)	0 (0)	3 (100)	0 (0)	6 (66.7)	0 (0)	3 (33.3)
TC	119 (74.8)	25 (21)	0 (0)	94 (79)	6 (27.3)	2 (9.1)	14 (63.6)	1 (16.7)	0 (0)	5 (83.3)	2 (66.6)	0 (0)	1 (33.3)	4 (44.4)	0 (0)	5 (55.6)
LNZ	4 (2.5)	115 (96.6)	1 (0.8)	3 (2.6)	22 (100)	0 (0)	0 (0)	5 (83.3)	0 (0)	1 (16.7)	3 (100)	0 (0)	0 (0)	9 (100)	0 (0)	0 (0)
Qui/Dal	108 (67.9)	2 (1.6)	0 (0)	117 (98.4)	22 (100)	0 (0)	0 (0)	1 (16.7)	0 (0)	5 (83.3)	0 (0)	0 (0)	3 (100)	9 (100)	0 (0)	0 (0)
TGC	0 (0.0)	119 (100)	0 (0)	0 (0)	22 (100)	0 (0)	0 (0)	6 (100)	0 (0)	0 (0)	3 (100)	0 (0)	0 (0)	9 (100)	0 (0)	0 (0)

S, Susceptible; I, Intermediate; R, Resistant. VAN, vancomicyn; AMP, ampicillin; PG, penicillin G; CIP, ciprofloxacin; LVX, levofloxacin; HLS, streptomycin; HLG, gentamicin; ERY, erythromycin; TC, tetracycline; LNZ, linezolid; Qui/Dal, quinupristin-dalfopristin; TGC, Tigecycline. ^a^ Odds Ratio = 6.8, *p* = 0.00. * Odds Ratio > 1, *p* = 0.00.

**Table 3:** Genes of glycopeptide resistance in enterococci.

Vancomycin-Resistant Enterococci Phenotype		Genotypes				
Species	MIC VAN (µg/mL)	*ddl*	*vanA*	*vanB*	*vanC_1_*	*vanC_2_*/*C_3_*
*E. faecalis*	32	+	+	−	−	−
*E. casseliflavus* (*n* = 3)	4	+	−	−	−	+
*E. gallinarum* (*n* = 5)	4	+	−	−	+	−
*E. gallinarum* (*n* = 1)	≥32	+	−	−	+	−
*E. faecium* (*n* = 6)	≥32	+	+	−	−	−
*E. faecium* (*n* = 1)	32	+	+	−	−	−

MIC VAN, Minimum inhibitory concentration vancomycin; *ddl,* genes encoding specific D-alanine—D-alanine ligases for *Enterococcus* spp.

**Table 4:** Prevalence of virulence factors in *Enterococcus* spp.

		*acm*		*asa*		*esp*		*cylA*		*gel E*		*agg*		*cylM*		*cylB*		*ccf*		*cpd*		*cob*	
Specimen (*n*)		*n*	(%)	*n*	(%)	*n*	(%)	*n*	(%)	*n*	(%)	*n*	(%)	*n*	(%)	*n*	(%)	*n*	(%)	*n*	(%)	*n*	(%)
Body fluids	88	23	(26.1)	44	(50.0)	35	(39.8)	9	(10.2)	55	(62.5)	4	(4.5)	8	(9.1)	8	(9.1)	72	(81.8)	51	(58.0)	12	(13.6)
Urine	44	7	(15.9)	24	(54.5)	19	(43.2)	8	(18.2)	33	(75.0)	1	(2.3)	4	(9.1)	4	(9.1)	40	(90.9)	35	(79.5)	8	(18.2)
Biopsy	16	2	(12.5)	12	(75.0)	6	(37.5)	2	(12.5)	9	(56.3)	1	(6.3)	2	(12.5)	1	(6.3)	15	(93.8)	12	(75.0)	3	(18.8)
Blood	6	2	(33.3)	4	(66.7)	1	(16.7)	0	(0.0)	5	(83.3)	0	(0.0)	0	(0.0)	0	(0.0)	4	(66.7)	3	(50.0)	1	(16.7)
Catheter	2	1	(50.0)	1	(50.0)	0	(0.0)	0	(0.0)	1	(50.0)	0	(0.0)	0	(0.0)	0	(0.0)	1	(50.0)	1	(50.0)	0	(0.0)
Other	3	1	(33.3)	1	(33.3)	1	(33.3)	0	(0.0)	2	(66.7)	0	(0.0)	0	(0.0)	0	(0.0)	3	(100.0)	1	(33.3)	0	(0.0)
TOTAL	159	36	(22.6)	86	(54.1)	62	(39.0)	19	(11.9)	105	(66.0)	6	(3.8)	14	(8.8)	13	(8.2)	135	(84.9)	103	(64.8)	24	(15.1)

*acm*, collagen-binding adhesin; *asa*, aggregation substance; *esp*, enterococcal surface protein; *cylA*, cytolysin A; *gel E*, gelatinase of *Enterococcus*; *agg*, aggregation substance; *cylM*, cytolysin M; *cylB*, cytolysin B; *ccf,* sex pheromone *ccf*; *cpd*, sex pheromone *cpd*; *cob*, sex pheromone *cob*.

**Table 5:** Prevalence of virulence factors in *Enterococcus* per specimen.

		*acm*		*asa*		*esp*		*cylA*		*gel E*		*agg*		*cylB*		*cylM*		*ccf*		*cob*		*cpd*	
	Total	*n*	(%)	*n*	(%)	*n*	(%)	*n*	(%)	*n*	(%)	*n*	(%)	*n*	(%)	*n*	(%)	*n*	(%)	*n*	(%)	*n*	(%)
	159	36	(22.6)	86	(54.1)	62	(39.0)	19	(11.9)	105	(66.0)	6	(3.8)	14	(8.8)	13	(8.2)	135	(84.9)	24	(15.1)	103	(64.8)
*E. faecalis*	119	7	(5.9) **^a^**	70	(58.8) **^a^**	50	(42.0)	6	(5.0)	87	(73.1) **^a^**	19	(16.0)	14	(11.8)	13	(10.9)	108	(90.8) **^a^**	21	(17.6)	94	(79.0) **^a^**
*E. faecium*	22	20	(90.9) **^a^**	4	(18.2) **^a^**	8	(36.4)	0	(0.0)	5	(22.7) **^a^**	0	(0.0)	0	(0.0)	0	(0.0)	15	(68.2) **^a^**	2	(9.1)	4	(18.2) **^a^**
*E. gallinarum*	6	2	(33.3)	3	(50.0)	2	(33.3)	0	(0.0)	4	(66.7)	0	(0.0)	0	(0.0)	0	(0.0)	3	(50.0)	1	(16.7)	2	(33.3)
*E. casseliflavus*	3	2	(66.7)	2	(66.7)	1	(33.3)	0	(0.0)	2	(66.7)	0	(0.0)	0	(0.0)	0	(0.0)	2	(66.7)	0	(0.0)	1	(33.3)
Others	9	5	(55.6)	7	(77.8)	1	(11.1)	0	(0.0)	7	(77.8)	0	(0.0)	0	(0.0)	0	(0.0)	7	(77.8)	0	(0.0)	2	(22.2)

*acm*, collagen-binding adhesin; *asa*, aggregation substance; *esp*, enterococcal surface protein; *cylA*, cytolysin A; *gel E*, gelatinase of *Enterococcus*; *agg*, aggregation substance; *cylM*, cytolysin M; *cylB*, cytolysin B; *ccf*, sex pheromone *ccf*; *cpd*, sex pheromone *cpd*; *cob*, sex pheromone *cob*. ^a^, Odds Ratio < 1 *p* < 0.05.

**Table 6:** Prevalence of virulence factors in *Enterococcus* spp. multidrug-resistant (MDR) and vancomycin-resistant enterococci (VREs).

Virulence Factors	MDR		No-MDR		Total		OR	CI 95%	*p*
	n	%	n	%	n	%			
	85	(53.5%)	74	(46.5%)	159	(100%)			
Adhesion	70	(82.3)	52	(70.2)	122	(76.7)			0.07
*acm*	27	(31.8)	9	(12.2)	36	(22.6)	3.3	1.4–7.7	0.00 *
Secretion	48	(56.4)	59	(79.7)	107	(67.3)	0.3	0.1–0.6	0.00 *
*gel E*	47	(55.3)	58	(78.4)	105	(66.0)	0.34	0.16–0.68	0.00 *
Aggregation	70	(82.3)	67	(90.5)	137	(86.1)			0.13
**Virulence factors**	**VRE**		**VSE**		**Total**		**OR**	**CI 95%**	***p***
	***n***	**%**	***n***	**%**	***n***	**%**			
	**17**	**(10.3%)**	**142**	**(89.7%)**	**159**	**(100%)**			
Adhesion	15	(88.2)	107	(75.3)	122	(76.7)			0.36
*acm*	11	(64.7)	25	(17.6)	36	(22.6)	8.5	2.9–25.3	0.00 *
Secretion	8	(47.0)	99	(69.7)	107	(67.3)			0.09
Sex pheromone	11	(64.7)	126	(88.7)	137	(86.1)	0.23	0.08–0.7	0.01 *
*ccf*	10	(58.8)	125	(88.0)	135	(84.9)	0.19	0.06–0.58	0.00 *
*cpd*	4	(23.5)	99	(69.7)	103	(64.7)	0.13	0.04–0.43	0.00 *

MDR, multidrug resistant; VRE, vancomycin-resistant *Enterococcus*; VSE, vancomycin-sensitive *Enterococcus*; *acm*, collagen-binding adhesin; *gel E*, gelatinase of *Enterococcus*; *ccf*, sex pheromone; *cpd*, sex pheromone. * OR: Odds Ratio; CI: Confidence interval.

**Table 7:** Prevalence of virulence factors in *Enterococcus* spp. according to glycopeptide resistance genotype.

Virulence Factors	*vanA*		No-*vanA*		Total		OR	CI 95%	*p*
	n	%	n	%	n	%			
	8	(5.1%)	151	(94.9%)	159	(100%)			
Adhesion	8	(100)	114	(75.5)	122	(76.7)			0.2
*acm*	7	(87.5)	29	(19.2)	36	(22.6)	29.4	3.5–248.9	0.00 *
Secretion	2	(25.0)	105	(69.5)	107	(67.3)	0.14	0.02–0.75	0.01 *
*gel E*	2	(25.0)	103	(68.2)	105	(66.0)	0.15	0.03–0.79	0.02 *
Sex pheromone	5	(62.5)	132	(87.4)	137	(86.1)			0.08
*cpd*	1	(12.5)	102	(67.5)	103	(64.7)	0.06	0.00–0.57	0.00 *
**Virulence factors**	***vanC***		**No-*vanC***		**Total**		**OR**	**CI 95%**	***p***
	***n***	**%**	***n***	**%**	***n***	**%**			
	**9**	**(38.4%)**	**150**	**(61.6%)**	**159**	**(100%)**			
Adhesion	7	(77.7)	115	(76.6)	122	(76.7)			1
Secretion	6	(66.6)	101	(67.3)	107	(67.3)			0.28
Sex pheromone	6	(66.6)	131	(87.3)	137	(86.1)			0.23
*ccf*	5	(55.5)	130	(90.0)	130	(86.6)	0.19	0.04–0.77	0.03 *

*vanA*, *vanA* glycopeptide resistance genotype; *vanC*, *vanC* glycopeptide resistance genotype; *acm*, collagen-binding adhesin; *gel E*, gelatinase of *Enterococcus*; *ccf*, sex pheromone; *cpd*, sex pheromone. * OR: Odds Ratio; CI: Confidence interval.

## Data Availability

The data used to support the findings of this study are restricted by the ethics board of the High Specialty Regional Hospital “Dr. Ignacio Morones Prieto” to protect patient privacy. Data are available from the corresponding author to this research, Edgar A. Turrubiartes-Martínez (edgar.turrubiartes@uaslp.mx), for researchers who meet the criteria for access to confidential data.

## Supplementary Materials

The following supporting information can be downloaded at: https://www.mdpi.com/article/10.3390/antibiotics14070663/s1: Table S1: Oligonucleotides used for species identification, resistance genes, and virulence factors.
