# Supplementary material for: Molecular Characterization of Vancomycin-Resistant Enterococcus spp. from Clinical Samples and Identification of a Novel Sequence Type in Mexico
Source: Antibiotics (Basel). 2025 Jun 30;14(7):663. doi: 10.3390/antibiotics14070663 (PMC12291894; doi:10.3390/antibiotics14070663)
Supplement: Supplementary file 1 [file antibiotics-14-00663-s001.zip › antibiotics-3618837-supplementary.pdf]

Supplementary Materials

Table S1. Oligonucleotides used for species identification, resistance genes, and virulence factors.

Oligonucleotide	Sequence 5′- 3′	pb
16S rRNA - F	GGATTAGATACCCTGGTAGTCC	320
16S rRNA - R	TCGTTGCGGGACTTAACCCAAC	
E. faecalis - F	ATCAAGTACAGTTAGTCTTTATTAG	941
E. faecalis - R	ACGATTCAAAGCTAACTGAATCAGT	
E. faecium - F	TTGAGGCAGACCAGATTGACG	658
E. faecium - R	TATGACAGCGACTCCGATTCC	
vanA - F	CATGAATAGAATAAAAGTTGCATA	1030
vanA - R	CCCCTTTAACGCTAATACGATCAA	
vanB - F	GTACAAACCGGGGCGAGGA	433
vanB - R	CCGCCATCCTCCTGCAAAAAA	
vanC1 - F	GGTATCAAGGAAACCTC	822
vanC1 - R	CTTCCGCCATCATAGCT	
vanC2/C3 - F	CGGGGAAGATGGCACTAT	484
vanC2/C3 - R	CGCAGGGACGGTGATTTT	
agg - F	AAGAAAAAGAAGTAGACCAAC	1533
agg - R	AAACGGCAAGACAAGTAAATA	
asa1 - F	GCACGCTATTACGAACTATGA	375
asa1 - R	TAAGAAAGAACATCACCACGA	
esp - F	AGATTTCATCTTTGATTCTTGG	510
esp - R	AATTGATTCTTTAGCATCTGG	
acm - F	GGCCAGAAACGTAACCGATA	135
acm - R	AACCAGAAGCTGGCTTTGTC	
cpd - F	TGGTGGGTTATTTTTCAATTC	782
cpd - R	TACGGCTCTGGCTTACTA	
cob - F	AACATTCAGCAAACAAAGC	1405
cob - R	TTGTCATAAAGAGTGGTCAT	
ccf - F	GGGAATTGAGTAGTGAAGAAG	543
ccf - R	AGCCGCTAAAATCGGTAAAAT	
hyl - F	ACAGAAGAGCTGCAGGAAATG	276
hyl - R	GACTGACGTCCAAGTTTCCAA	
gelE - F	TATGACAATGCTTTTTGGGAT	213
gelE - R	AGATGCACCCGAAATAATATA	
cylA - F	ACTCGGGGATTGATAGGC	688
cylA - R	GCTGCTAAAGCTGCGCTT	
cylM - F	CTGATGGAAAGAAGATAGTAT	742
cylM - R	TGAGTTGGTCTGATTACATTT	
cylB - F	ATTCCTACCTATGTTCTGTTA	843

cylB - R	AATAAACTCTTCTTTTCCAAC	
